# Current Advances and Future Directions for Sensitizing Gastric Cancer to Immune Checkpoint Inhibitors

**DOI:** 10.1002/cam4.71065

**Published:** 2025-07-21

**Authors:** Wenke Li, Menghui Xu, Mo Cheng, Jing Wei, Lin Zhu, Yu Deng, Fukun Guo, Feng Bi, Ming Liu

**Affiliations:** ^1^ Gastric Cancer Center/Cancer Center, West China Hospital, Sichuan University Chengdu Sichuan China; ^2^ Department of Integrated Traditional and Western Medicine West China Hospital, Sichuan University Chengdu China; ^3^ School of Basic Medical Sciences Chengdu University Chengdu Sichuan China; ^4^ Division of Experimental Hematology and Cancer Biology Cincinnati Children's Hospital Medical Center Cincinnati Ohio USA; ^5^ Abdominal Oncology Ward, Cancer Center, West China Hospital, Sichuan University Chengdu China

**Keywords:** gastric cancer, immunotherapy, immunotherapy sensitization, PD‐1/PD‐L1 inhibitors

## Abstract

**Background:**

Immunotherapy combined with chemotherapy has become the standard treatment for HER2‐negative gastric cancer (GC), but its clinical benefits remain limited, with a median progression‐free survival (mPFS) of 6–8 months and median overall survival (mOS) of 15–18 months. These outcomes are particularly poor in patients with CPS < 1. The marked heterogeneity of GC, along with primary and secondary resistance, presents significant clinical challenges and underscores the urgent need for novel therapeutic strategies.

**Recent Advances:**

To address these limitations, several combination therapies are being explored. Anti‐VEGF therapy combined with immune checkpoint inhibitors (ICIs) has shown synergistic effects by enhancing immune cell infiltration and reducing tumor‐mediated immunosuppression, thereby improving response rates and survival. Radiotherapy combined with ICIs also holds promise, with low‐dose radiation remodeling the tumor microenvironment and high‐dose radiation inducing immunogenic cell death. Other potential combinations include PD‐1/PD‐L1 inhibitors paired with targeted therapies against HER2, FGFR2, DKK1, PARP, LSD1, HDAC, and other emerging targets. Novel approaches such as hyperbaric oxygen therapy, oncolytic viruses, metabolic modulators, and fecal microbiota transplantation are also under investigation to further enhance immune responses.

**Conclusion:**

These multimodal strategies represent a promising shift toward personalized, mechanism‐driven immunotherapy sensitization. By targeting diverse pathways to overcome immune resistance, they aim to reshape the tumor microenvironment, restore immune responsiveness, and improve outcomes in GC. While many remain in early‐stage development, accumulating evidence supports their potential. Future research should prioritize optimizing combination regimens, clarifying resistance mechanisms, and identifying predictive biomarkers through multi‐omics and artificial intelligence to enable more precise, individualized immunotherapy.

## 1 Introduction

GC ranks as the fifth most common malignancy and the fifth leading cause of cancer‐related death worldwide [1]. Owing to its insidious onset, most patients are diagnosed at an advanced stage, leading to a poor overall prognosis. Historically, chemotherapy has been the mainstay treatment for advanced or unresectable GC. In recent years, with the successful application of ICIs in various malignancies, chemotherapy combined with ICIs has become the first‐line therapy for advanced or unresectable GC.

Immune checkpoints act as “brakes” in cellular immune responses. Under normal circumstances, they negatively regulate the immune system to maintain self‐tolerance and prevent autoimmune diseases [2]. However, some tumors exploit this mechanism to evade recognition and attack by the immune system, leading to immune escape. ICIs can bind to their corresponding molecules, inhibiting immune checkpoints and reversing the immunosuppressive state in the tumor microenvironment (TME), thereby exerting antitumor effects. Among ICIs, programmed cell death protein 1 (PD‐1) and programmed death‐ligand 1 (PD‐L1) inhibitors are the most commonly used.

The combination of ICIs, such as nivolumab, with chemotherapy has significantly improved the efficacy in advanced or unresectable GC, but many challenges remain [3]. These challenges are, in part, attributable to the significant molecular heterogeneity of GC. Based on comprehensive genomic analyses, such as The Cancer Genome Atlas (TCGA), GC can be classified into distinct molecular subtypes, including Epstein–Barr virus‐positive (EBV+), microsatellite instability‐high (MSI‐H), genomically stable (GS), and chromosomally unstable (CIN) [4]. These subtypes exhibit varied TMEs, immunogenic profiles, and importantly, differential responses to immunotherapy. For instance, EBV+ and MSI‐H subtypes often present with higher tumor mutational burden (TMB) and PD‐L1 expression, rendering them more responsive to ICIs. In contrast, the GS and CIN subtypes, which constitute the majority of GCs, are frequently less immunogenic and demonstrate limited benefit from ICI monotherapy, thereby highlighting the pressing need for innovative strategies to sensitize these tumors to ICIs [5, 6].

Furthermore, resistance to PD‐1/PD‐L1 inhibitors, whether primary or acquired, remains a significant challenge [7]. Therefore, there is an urgent need for new therapeutic strategies in clinical practice to further increase the efficacy of immunotherapy. In light of this issue, this review summarizes the current novel strategies to increase the sensitivity of GC to ICIs. A comprehensive overview of these strategies, detailing their mechanisms, current research stages, evidence levels, and key challenges, is presented in Table 1.

**TABLE 1:** Summary of research stages and evidence levels for combination strategies in GC/GEJC.

Combination strategy	Mechanism of synergy	Current research stage		Level of evidence	Key challenges
		Available	Ongoing		
1. Chemotherapy + PD‐1/PD‐L1 Inhibitors					
	ICD, TME remodel, ↑Antigenicity, ↓Immunosuppressive cells	Phase III clinical trial	Further optimization (multiple phase II/III trials)	NCCN, ESMO, CSCO guidelines recommended (HER2^−^, PD‐L1 CPS ≥ 1, or MSI‐H/dMMR)	Optimal chemo partner, dosage, duration.
2. Radiotherapy + PD‐1/PD‐L1 Inhibitors					
CFRT	ICD, TME remodel, Abscopal effect	Phase II clinical trial	—	Promising	Optimal dose/fractionation with ICI, AEs.
SBRT	Similar to CFRT	Preclinical research	—	Exploratory	Lack of clinical data in GC/GEJC.
LDRT	TME remodel	Preclinical research	Phase Ib/II clinical trial	Exploratory	Lack of clinical data in GC/GEJC.
SFRT	Heterogeneous dose, activate multiple immune mechanisms	Preclinical research	Phase I clinical trial	Exploratory	Lack of clinical data in GC/GEJC.
3. Dual Immune Checkpoint Blockade					
CTLA‐4	Distinct T‐cell activation stages	Phase III clinical trial	Phase III clinical trial	CSCO guideline recommended	High risk of irAEs, balancing efficacy/toxicity.
LAG‐3	Overcome compensatory LAG‐3 upregulation, reinvigorate T‐cells	Phase II clinical trial^a^	Phase II clinical trial	Exploratory	Lack of demonstrated efficacy in GC/GEJC so far, need for predictive biomarkers.
TIM‐3	Restore exhausted T‐cells, overcome resistance	Preclinical research	Phase Ib/II clinical trial (including GC/GEJC cohort)	Exploratory	Lack of clinical data in GC/GEJC
TIGIT	Restore CD226 co‐stimulation, enhance T/NK cells activity	Phase II clinical trial	Phase III clinical trial	Promising	Need for Phase III validation in GC/GEJC, identifying optimal biomarkers, long‐term outcomes.
LILRB1	Modulate myeloid cells (TAMs), activate T/NK cells	Preclinical research	Phase Ib/II clinical trial (Solid tumor including GC/GEJC)	Exploratory	Lack of clinical data in GC/GEJC.
4. Anti‐VEGF/VEGFR + PD‐1/PD‐L1 Inhibitors					
	Normalize vasculature, ↓Immunosuppression, ↑Immune cell infiltration	Phase III clinical trial^b^	Phase III clinical trial	CSCO guideline recommended (AFP‐GC)	Need for Phase III validation in GC/GEJC, identifying optimal biomarkers (e.g., AFP status, angiogenesis markers).
5. HER2‐Targeted Therapy + PD‐1/PD‐L1 Inhibitors					
	Adaptive ↑PD‐L1, Enhance DC presentation, Synergistic immune modulation	Phase III clinical trial	Phase III clinical trial	NCCN, ESMO, CSCO guidelines recommended (HER2^+^, PD‐L1 CPS ≥ 1)	Patient selection refinement (beyond HER2/PD‐L1, novel biomarkers); Combined toxicity management (irAEs, specific anti‐HER2 AEs).
6. FGFR2‐Targeted Therapy + PD‐1/PD‐L1 Inhibitors					
	↑CD8^+^ T‐cell infiltration by inhibiting FGFR2 signaling	Preclinical research	Phase II clinical trial (Solid tumor including GC/GEJC)	Exploratory	Lack of clinical data in GC/GEJC.
7. DKK1‐Targeted Therapy + PD‐1/PD‐L1 Inhibitors					
	Reprogram TAMs (M2 to M1), ↑Effector cell infiltration	Phase II clinical trial	—	Promising	Validation in Phase III trials; Confirming DKK1 as a predictive biomarker; Long‐term efficacy and safety data.
8. ADCs + PD‐1/PD‐L1 Inhibitors					
	ICD, Bystander effect, ↑Neoantigen release	Phase I/II clinical trial	Phase III clinical trial	Promising	Managing overlapping and potentially synergistic toxicities.
9. Epigenetic & DNA Repair Regulators + PD‐1/PD‐L1					
PARP Inhibitors	↑TMB, ↑Neoantigen release, cGAS‐STING activation, Adaptive ↑PD‐L1	Preclinical research	Phase I/II clinical trial	Exploratory	Lack of definitive GC/GEJC‐specific efficacy data, Managing combined toxicities.
LSD1 Inhibitors	↓PD‐L1^+^ exosomes, restore T‐cell response	Preclinical research	—	Highly Experimental	Lack of clinical data in GC/GEJC.
HDAC Inhibitors	↑MHC expression, enhance antigen presentation, Adaptive ↑PD‐L1	Preclinical research	—	Highly Experimental	Lack of clinical data in GC/GEJC.
10. Other Combination Therapies					
FMT	Modulate gut microbiome, ↑beneficial bacteria, improve systemic immune tone	Preclinical research	—	Highly Experimental	Lack of clinical data in GC/GEJC.
ACT	Enhance reinfused T‐cell function	Preclinical research	Phase II clinical trial	Exploratory	Lack of clinical data in GC/GEJC.
OVs	TME remodel, ↑Neoantigen release, Adaptive ↑PD‐L1	Preclinical research	—	Highly Experimental	Lack of clinical data in GC/GEJC.
Co‐stimulatory Receptor Agonists	Enhance T‐cell activation/proliferation	Preclinical research	—	Highly Experimental	Lack of clinical data in GC/GEJC.
HBOT	Reduce ECM density, improve drug penetration, Reverse hypoxic TME	Preclinical research	Phase Ib/II clinical trial	Exploratory	Lack of clinical data in GC/GEJC.

Abbreviations: ACT, Autologous Cell Therapy; ADCs, Antibody‐Drug Conjugates; AEs, Adverse Events; AFP‐GC, Alpha‐Fetoprotein producing Gastric Cancer; CFRT, Conventional Fractionated Radiotherapy; cGAS‐STING, cyclic GMP‐AMP Synthase/Stimulator of Interferon Genes; CPS, Combined Positive Score; CSCO, Chinese Society of Clinical Oncology; CTLA‐4, Cytotoxic T‐lymphocyte‐associated protein 4; DC, Dendritic Cell; DKK1, Dickkopf‐related protein 1; dMMR, deficient Mismatch Repair; ECM, Extracellular Matrix; ESMO, European Society for Medical Oncology; FGFR2, Fibroblast Growth Factor Receptor 2; FMT, Fecal Microbiota Transplantation; GC, Gastric Cancer; GEJC, Gastroesophageal Junction Cancer; HBOT, Hyperbaric Oxygen Therapy; HDAC, Histone Deacetylase; HER2, Human Epidermal growth factor Receptor 2; ICD, Immunogenic Cell Death; irAEs, immune‐related Adverse Events; LAG‐3, Lymphocyte‐activation gene 3; LDRT, Low‐Dose Radiotherapy; LILRB1, Leukocyte Immunoglobulin‐like Receptor B1; LSD1, Lysine‐Specific Demethylase 1; MHC, Major Histocompatibility Complex; MSI‐H, Microsatellite Instability‐High; NCCN, National Comprehensive Cancer Network; NK cells, Natural Killer cells; OVs, Oncolytic Viruses; PD‐1, Programmed cell Death protein 1; PD‐L1, Programmed Death‐Ligand 1; SBRT, Stereotactic Body Radiotherapy; SFRT, Spatially Fractionated Radiation Therapy; TAMs, Tumor‐Associated Macrophages; TIGIT, T‐cell immunoreceptor with Ig and ITIM domains; TIM‐3, T‐cell Immunoglobulin and Mucin‐domain containing‐3; TMB, Tumor Mutational Burden; TME, Tumor Microenvironment; VEGF, Vascular Endothelial Growth Factor; VEGFR, Vascular Endothelial Growth Factor Receptor.

^a^ The RELATIVITY‐060 trial did not meet its primary efficacy endpoints.

^b^ The LEAP‐015 trial demonstrated a statistically significant improvement in PFS but did not meet its primary endpoint of OS.

## 2 Mechanisms of PD‐1/PD‐L1 Inhibitors in Antitumor Activity

The process by which immune cells eliminate cancer cells, known as the cancer‐immunity cycle, involves several stages: antigen recognition, T‐cell activation, T‐cell recruitment, and tumor cell killing [8, 9, 10]. However, tumors often evade immune surveillance by exploiting immune checkpoints, with the PD‐1/PD‐L1 axis being the most extensively studied and utilized. PD‐1 on activated T cells binds its ligands (PD‐L1/PD‐L2), often expressed on antigen‐presenting cells and cancer cells [2]. When PD‐L1 on tumor cells interacts with PD‐1 on T cells, it activates downstream signaling pathways that inhibit T‐cell signaling and function, suppress T‐cell proliferation and activation, and diminish immune‐stimulating factors, ultimately leading to immune evasion [11].

Blocking the PD‐1/PD‐L1 pathway can restore immune surveillance, enabling immune cells to respond more effectively to tumors. However, resistance to these therapies, which can be primary (intrinsic lack of response from the outset) or acquired (loss of initial response over time), remains a significant barrier for many patients. Primary resistance to immunotherapy is frequently associated with “cold” tumors characterized by poor immunogenicity from reduced antigen presentation and limited T cell infiltration due to defective trafficking or an adverse chemokine profile [12]. Acquired resistance, on the other hand, can develop in initially responsive tumors through various adaptive mechanisms. These include the loss or downregulation of PD‐L1 expression on tumor cells, the emergence of mutations in IFN signaling pathway components (e.g., Janus Kinase 1/2 mutations) which impair antigen presentation machinery and T cell effector functions, or the upregulation of alternative immune checkpoints leading to persistent T cell exhaustion [13, 14]. Furthermore, the accumulation of immunosuppressive cells in the TME, such as M2‐polarized macrophages, myeloid‐derived suppressor cells (MDSCs), and fibroblasts, can secrete inhibitory factors that further weaken T‐cell activity and contribute to both primary and acquired resistance [15, 16, 17].

This landscape of inherent and acquired resistance mechanisms poses a particular challenge for the majority of GC subtypes, which, unlike highly immunogenic MSI‐H or EBV+ tumors, derive limited benefit from ICI monotherapy. Therefore, there is an urgent need to explore novel therapeutic strategies to counteract tumor immune escape pathways and enhance the efficacy of PD‐1/PD‐L1 inhibitors.

## 3 Strategies to Increase Sensitivity to PD‐1/PD‐L1 Inhibitors

**FIGURE 1:**
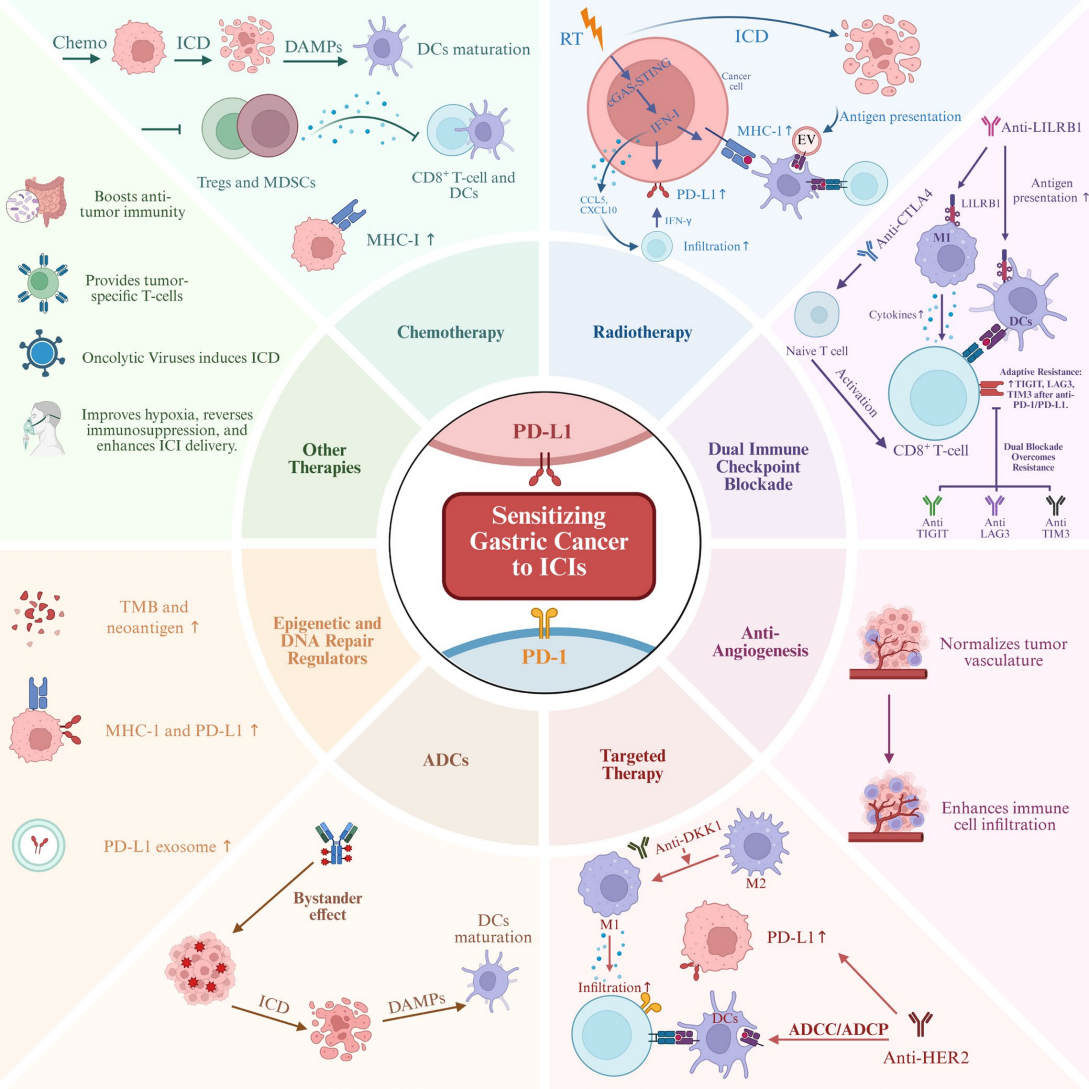
Synergistic Mechanisms of PD‐1/PD‐L1 Inhibitor Combinations. This figure demonstrates how various combination therapies enhance the efficacy of PD‐1/PD‐L1 inhibitors in GC. Chemotherapy contributes to synergy by inducing ICD, which activates DCs and promotes their maturation for enhanced antigen presentation. It also upregulates MHC‐I molecule expression and modulates immune cell populations within the TME. Radiotherapy induces ICD and antigen release, activating DCs. Radiation also activates the cGAS‐STING pathway, leading to IFN‐I production. IFN‐I upregulates MHC‐I expression, promotes antigen presentation, increases CXCL10/CCL5 chemokine expression thereby enhancing CD8^+^ T‐cell infiltration, and can upregulate PD‐L1 via IFN‐γ signaling. Dual Immune Checkpoint Blockade involves multiple mechanisms: Anti‐CTLA‐4 antibodies facilitate initial T‐cell activation; anti‐LILRB1 antibodies can disinhibit antigen‐presenting cells. Post‐antiPD‐1/PD‐L1 therapy, adaptive resistance can manifest as upregulated TIGIT, LAG‐3, and TIM‐3 expression; co‐blockade of these targets with PD‐1/PD‐L1 restores exhausted T‐cell function. Anti‐Angiogenic Therapy normalizes tumor vasculature, improving immune cell infiltration into the TME. Targeted Therapies, such as anti‐DKK1 and anti‐HER2 agents, also induce immunosensitization. Anti‐DKK1 therapy promotes M2‐to‐M1 macrophage polarization and enhances CD8^+^ T‐cell infiltration. Anti‐HER2 therapies facilitate DC‐mediated antigen presentation via ADCC/ADCP and can also induce adaptive PD‐L1 upregulation. ADCs operate by delivering cytotoxic payloads to tumor cells, causing targeted cell death and a bystander effect on adjacent antigen‐negative cells, which leads to ICD, DAMP release, and DC maturation. Epigenetic and DNA Repair Regulators can upregulate MHC‐I and PD‐L1 expression, increase TMB and neoantigen release, and modulate PD‐L1‐containing exosomes, thereby augmenting tumor immunogenicity. Other Emerging Therapies demonstrate varied mechanisms: HBOT improves hypoxia, reverses immunosuppression, and enhances T‐cell function and ICI penetration. FMT modulates the gut microbiome, promoting systemic anti‐tumor immunity and ICI responsiveness. OVs induce ICD and activate both innate and adaptive immune responses. ACT directly provide tumor‐specific effector T‐cells. Collectively, these diverse strategies modify the TME and tumor cell characteristics to enhance the anti‐tumor activity of PD‐1/PD‐L1 inhibitors in GC. Created in https://BioRender.com. ACT, Autologous Cell Therapy; ADCC, Antibody‐Dependent Cellular Cytotoxicity; ADCP, Antibody‐Dependent Cellular Phagocytosis; ADCs, Antibody‐Drug Conjugates; CCL5, Chemokine (C‐C motif) Ligand 5; cGAS‐STING, cyclic GMP‐AMP Synthase/Stimulator of Interferon Genes (pathway); CTLA‐4, Cytotoxic T‐lymphocyte‐associated protein 4; CXCL10, Chemokine (C‐X‐C motif) Ligand 10; DAMP, Damage‐Associated Molecular Pattern; DKK1, Dickkopf‐related protein 1; FMT, Fecal Microbiota Transplantation; HBOT, Hyperbaric Oxygen Therapy; HER2, Human Epidermal growth factor Receptor 2; ICD, Immunogenic Cell Death; IFN‐I, Type I Interferon; LAG‐3, Lymphocyte‐activation gene 3; LILRB1, Leukocyte Immunoglobulin‐like Receptor B1; OVs, Oncolytic Viruses; TIM‐3, T‐cell Immunoglobulin and Mucin‐domain containing‐3; TIGIT, T‐cell immunoreceptor with Ig and ITIM domains; TMB, Tumor Mutational Burden; VEGF, Vascular Endothelial Growth Factor.

Several distinct strategies are being explored to enhance the sensitivity of GC/GEJC to PD‐1/PD‐L1 inhibitors, largely by modulating the tumor immune microenvironment and overcoming resistance pathways. Figure 1 illustrates the key synergistic mechanisms by which various combination therapies, discussed in this section, can potentiate the efficacy of PD‐1/PD‐L1 inhibitors.

### 3.1 Chemotherapy in Combination With PD‐1/PD‐L1 Inhibitors

Chemotherapy can enhance the antitumor effects of PD‐1/PD‐L1 inhibitors through various mechanisms. First, many chemotherapy drugs, such as paclitaxel and cisplatin, can increase the antigenicity of tumor cells and induce immunogenic cell death (ICD), a form of regulated cell death that triggers an adaptive immune response by releasing damage‐associated molecular patterns (DAMPs) [18, 19, 20, 21]. During the ICD process, dying cells release DAMPs, which bind to pattern recognition receptors (PRRs) on the surface of DCs, promoting DC maturation and T‐cell activation [22]. Additionally, gemcitabine can enhance the immune system's ability to recognize and kill tumor cells by reducing the infiltration of immunosuppressive cells such as regulatory T cells (Tregs) and myeloid‐derived suppressor cells (MDSCs) and increasing the expression of MHC class I molecules [23].

Several large clinical studies, including KEYNOTE‐859 [24], CHECKMATE‐649 [25], and ORIENT‐16 [26], have evaluated the efficacy of chemotherapy combined with PD‐1/PD‐L1 inhibitors in patients with HER2‐negative advanced GC/GEJC. The results consistently demonstrated better outcomes than those of chemotherapy alone. On the basis of these findings, chemotherapy combined with immunotherapy has become the first‐line treatment choice for HER2‐negative advanced GC/GEJC. However, the efficacy of combining PD‐1 inhibitors with different chemotherapy regimens in the treatment of GC/GEJC remains unclear. A meta‐analysis comparing 23 first‐line treatment regimens in HER2‐negative advanced GC demonstrated that, compared to the PF regimen (cisplatin and fluorouracil), the combination of XELOX (Capecitabine and Oxaliplatin) with sintilimab (a PD‐1 inhibitor) or SOX (S‐1 and Oxaliplatin) with nivolumab (another PD‐1 inhibitor) significantly improved overall survival (OS) [27]. Therefore, exploring the efficacy of different chemotherapy regimens in combination with PD‐1 inhibitors is highly relevant for GC treatment.

Furthermore, the dosage and frequency of chemotherapy may impact the efficacy of ICIs, and the optimal chemotherapy dose remains under investigation. Researchers have launched a phase II clinical trial exploring metronomic chemotherapy combined with a PD‐1 inhibitor for the treatment of breast cancer. Compared with conventional chemotherapy, the metronomic VEX regimen (cyclophosphamide, capecitabine, and vinorelbine) significantly improved the disease control rate (DCR) and progression‐free survival (PFS). Further analysis indicated that metronomic chemotherapy, as opposed to traditional dosing, may better remodel the immune microenvironment. The metronomic VEX regimen notably reduced the proportion of peripheral blood CD4^+^ central memory T cells while increasing the number of CD4^+^ CD11b^+^ CD28^−^ T cells, which are closely associated with the response to ICIs. Additionally, an increase in monocyte and natural killer (NK) cell proportions was observed, suggesting an immune‐modulatory effect of metronomic chemotherapy [28]. While promising, the role of metronomic chemotherapy in combination with ICIs for GC is still under active investigation, with efficacy and optimal regimens yet to be fully established. A clinical trial is currently underway combining PLOF (paclitaxel, oxaliplatin, and 5‐fluorouracil) metronomic chemotherapy with sintilimab as neoadjuvant treatment for locally advanced GC (NCT06054906).

### 3.2 Radiotherapy in Combination With PD‐1/PD‐L1 Inhibitors

Radiotherapy is a cornerstone of cancer treatment and is widely employed across diverse tumor types. Advances in radiotherapy have clarified its antitumor mechanisms, leading to the development of novel modalities, including conventional fractionated radiotherapy (CFRT), hypofractionated radiotherapy (HFRT), stereotactic body radiotherapy (SBRT), and low‐dose‐rate radiotherapy (LDRT). Beyond its direct cytotoxic effects, radiotherapy can stimulate tumor‐associated immune responses through the induction of ICD [29, 30, 31]. In addition to increasing neoantigen exposure, radiation can reprogram the immunosuppressive TME into an immunostimulatory microenvironment by upregulating MHC expression on cancer cells, promoting antigen presentation, and inducing T‐cell proliferation and activation [32, 33]. Radiotherapy also reduces the number of local immunosuppressive factors, such as Tregs and MDSCs [34], transforming tumors from an “immune‐cold” state to an “immune‐active” state, which increases effector T‐cell infiltration and activation even in low‐immunogenic tumors [35]. However, the mechanisms behind combining different radiotherapy modalities with ICIs differ slightly. In some cases, compared to SBRT, CFRT may induce vascular damage at the irradiated site, leading to hypoxia and the formation of an immunosuppressive TME. Therefore, the optimal strategies for combining different radiotherapy modalities with PD‐1/PD‐L1 inhibitors remain a topic of ongoing investigation.

Studies have demonstrated that radiotherapy can induce regression of local tumors while also reducing the size of distant, non‐irradiated tumors through enhanced systemic immune activation—a phenomenon known as the “abscopal effect”. This effect is particularly pronounced when radiotherapy is combined with immunotherapy, indicating a synergistic interaction, although the precise mechanisms remain unclear [36]. Radiotherapy can induce upregulation of PD‐L1 expression in the TME, which may reflect an adaptive immune resistance. When combined with PD‐1/PD‐L1 blockade, this may enhance antitumor immunity and improve therapeutic outcomes [37, 38]. Hypoxia is a key contributor to radioresistance in tumors. However, PD‐1/PD‐L1 inhibitors have been shown to normalize tumor vasculature and alleviate hypoxia within the tumor microenvironment, thereby increasing tumor cell sensitivity to radiotherapy [39].

In the treatment of GC/GEJC, a phase II clinical trial assessed the efficacy and safety of pembrolizumab combined with CFRT as neoadjuvant therapy for locally advanced cases. The results revealed that 35.7% of patients achieved a pathologic complete response (pCR), outperforming historical comparisons of chemotherapy combined with radiotherapy as neoadjuvant therapy [40]. While numerous studies have confirmed the synergistic effects of combining radiotherapy with PD‐1/PD‐L1 inhibitors, the optimal radiation dose remains unclear. High‐dose radiotherapy can promote antigen release and T‐cell activation, but it is also associated with a higher incidence of adverse reactions [41]. The TME in metastatic lesions is often rich in fibroblasts, MDSCs, and Tregs, which can hinder the penetration of PD‐1 inhibitors and the infiltration of immune effector cells, thereby reducing the efficacy of immunotherapy [42]. Research has shown that LDRT (usually less than 10 Gy) can reprogram the suppressive stroma around tumors, reversing the immunosuppressive microenvironment with fewer adverse effects [43]. LDRT combined with PD‐1/PD‐L1 inhibitors may lead to better outcomes in patients with GC, but clinical evidence remains limited. Our team is conducting a neoadjuvant clinical study for GC aimed at exploring the optimal radiation dose when combined with chemotherapy and immunotherapy (NCT06266871).

Spatially fractionated radiation therapy (SFRT) is a distinctive radiation treatment technique that activates the immune system and enhances the tumor's immune response by creating heterogeneous dose distributions within the tumor target area [44]. Some researchers suggest that varying radiation doses influence the immune system through different pathways. High doses promote ICD, which facilitates the release of tumor‐specific antigens; intermediate doses increase the expression of immune susceptibility markers and activate type I IFN responses; and low doses promote the secretion of inflammatory cytokines, which modulate immune cell infiltration and activation. By combining high‐ and low‐dose radiation to induce intratumoral dose heterogeneity, the antitumor immune response can be significantly enhanced through the concurrent activation of multiple immune mechanisms. Compared to uniform‐dose radiation, heterogeneous‐dose radiation promotes the clonal expansion of effector CD8^+^ T cells and supports the development of memory cells, potentially fostering more durable immune memory [45]. Currently, we are conducting a phase I clinical trial (ChiCTR2400089068) to explore the safety, tolerability, and preliminary efficacy of LDRT and SBRT in combination with CAPOX (capecitabine and oxaliplatin) and toripalimab as a first‐line treatment for HER2‐negative advanced GC/GEJC.

### 3.3 Dual Immune Checkpoint Blockade

Blocking one immune checkpoint can lead to compensatory upregulation of other receptors on T cells, potentially causing resistance to PD‐1/PD‐L1 inhibitors and highlighting the rationale for dual or multiple checkpoint blockade [46]. Consequently, exploring combinations of dual or multiple ICIs presents significant therapeutic potential.

#### 3.3.1 CTLA‐4 Inhibitors in Combination With PD‐1/PD‐L1 Inhibitors

Cytotoxic T lymphocyte‐associated protein 4 (CTLA‐4) and PD‐1 are two critical immune checkpoints that play essential roles in regulating immune self‐tolerance and tumor immune evasion. CTLA‐4 primarily inhibits T‐cell activation during the initial immune response by competitively binding to the costimulatory ligands B7‐1 and B7‐2, thereby preventing excessive autoimmune reactions [8]. In contrast, PD‐1 functions mainly in peripheral tissues by binding to its ligands PD‐L1/PD‐L2, inhibiting the effector functions of already activated T cells, and acting during the later stages of the immune response [47].

Due to their distinct mechanisms and sites of action, anti‐PD‐1 therapy alone often yields limited effects in “cold tumors” that lack CD8^+^ T‐cell infiltration, as there are insufficient effector T cells to mediate antitumor responses [48]. Anti‐CTLA‐4 therapy promotes naïve T‐cell activation and proliferation in lymph nodes, generating effector CD8^+^ T cells that can migrate to the TME and exert antitumor effects [8]. Thus, combining anti‐CTLA‐4 and anti‐PD‐1 therapies can enhance overall antitumor immunity, especially in low T‐cell infiltration tumors, potentially overcoming monotherapy limitations and improving outcomes.

The combination of anti‐PD‐1 and anti‐CTLA‐4 therapies has shown favorable efficacy in various cancers, such as melanoma, cervical cancer, and lung cancer [49, 50, 51], where it can significantly prolong patient PFS and OS compared with PD‐1 inhibitors alone, while also exhibiting manageable safety profiles. Initial explorations with ipilimumab and nivolumab in GC/GEJC (CheckMate‐032) indicated some antitumor activity [52], with related phase III trials ongoing (NCT05144854). However, the phase II Moonlight trial (AIO‐STO‐0417) found that adding PD‐1/CTLA4 dual immunotherapy to first‐line FOLFOX chemotherapy failed to improve PFS or OS, while significantly increasing Grade ≥ 3 toxicities—raising concerns about the risk–benefit ratio and limiting its clinical applicability [53]. Nevertheless, this combination strategy has demonstrated remarkable success in specific contexts. In the neoadjuvant treatment of dMMR (deficient mismatch repair) GC patients, studies such as GERCOR NEONIPIGA and INFNITY, using PD‐1/PD‐L1 inhibitors with CTLA‐4 antibodies, reported impressive pCR rates of 59% and 60%, respectively, suggesting this dual approach is becoming a new choice for this population [54, 55].

A promising development is the PD‐1/CTLA‐4 bispecific antibody, cadonilimab [56]. Notably, the phase III COMPASSION‐15 trial assessed first‐line cadonilimab combined with chemotherapy in patients with advanced GC. The combination therapy group demonstrated significant improvements in mOS (14.1 vs. 11.1 months), mPFS (7.0 vs. 5.3 months), and ORR (65.2% vs. 48.9%) compared to the chemotherapy‐alone group. The incidence of grade ≥ 3 adverse events was higher in the combination therapy group (73.8% vs. 63.2%), but remained within a manageable range [57]. Based on these findings, cadonilimab combined with chemotherapy is now recommended by the CSCO (chinese society of clinical oncology) guidelines as a first‐line treatment option for advanced GC, regardless of PD‐L1 expression. Furthermore, cadonilimab's potential extends to patients who have progressed on prior PD‐1/PD‐L1 inhibitor‐based therapy. The AK109‐201 phase Ib/II study evaluated cadonilimab combined with pulocimab (an anti‐VEGFR2 mAb) and paclitaxel versus pulocimab plus paclitaxel in this setting. The addition of cadonilimab led to improvements in ORR (48.0% vs. 39.3%), median duration of response (5.5 vs. 4.2 months), mPFS (6.8 vs. 5.5 months), and mOS (12.9 vs. 8.9 months) [58]. A phase III trial (NCT06341335) is currently underway to further evaluate this regimen.

#### 3.3.2 LAG‐3 Inhibitors in Combination With PD‐1/PD‐L1 Inhibitors

Lymphocyte‐activation gene 3 (LAG‐3), an immune checkpoint receptor expressed on activated T cells (both CD4^+^ and CD8^+^), Tregs, NK cells, and B cells, plays a crucial role in regulating immune suppression [59]. LAG‐3 primarily binds to MHC class II molecules on antigen‐presenting cells and Fibrinogen‐Like Protein 1 (FGL1) expressed by tumor cells. Its interaction with peptide–MHC class II (pMHC‐II) competitively disrupts CD4 binding, suppressing T cell activation [60]. Furthermore, the interaction between LAG‐3 and FGL1 may lead to reduced IL‐2 production within the TME, further inhibiting T cell function [61]. Interestingly, the prognostic value of LAG‐3 expression in GC is heterogeneous; while some studies associate high LAG‐3 infiltration with an immunoevasive contexture and poorer outcomes in specific molecular subtypes (e.g., EBV‐positive or MLH1‐defective GC) [62], other research suggests it may correlate with improved survival, highlighting the complexity of its role [63, 64]. LAG‐3 frequently co‐expresses with PD‐1 on exhausted T cells within the GC TME, and this dual expression often signifies a more profound state of T cell dysfunction than the expression of either receptor alone. Crucially, resistance or suboptimal response to PD‐1/PD‐L1 blockade can be associated with the compensatory upregulation of LAG‐3 on T cells, positioning LAG‐3 as a key target to overcome ICI resistance [60].

The combination of relatlimab (an anti‐LAG‐3 antibody) and nivolumab has shown promising efficacy in advanced melanoma, with patients achieving significantly better outcomes than those receiving nivolumab monotherapy, highlighting the potential of this dual immunotherapy approach [65]. However, this success did not translate to GC. In the RELATIVITY‐060 trial evaluating nivolumab, relatlimab, and chemotherapy in advanced GC/GEJC, the combination failed to meet its primary efficacy endpoints. Notably, the addition of relatlimab did not improve outcomes compared to nivolumab plus chemotherapy alone in the overall LAG‐3 ≥ 1% population and even showed trends toward numerically lower response rates and survival. Moreover, the triple regimen was associated with a higher incidence of grade 3/4 treatment‐related adverse events and more frequent treatment discontinuations due to toxicity. Subgroup analysis suggested a potential benefit in patients whose tumors expressed LAG‐3 at ≥ 5%, but these findings require validation in future studies [66]. Additionally, AK129, a bispecific antibody targeting both PD‐1 and LAG‐3, is currently being evaluated in a clinical study to assess its efficacy and safety in patients with unresectable locally advanced or metastatic GC/GEJC (NCT06586294).

#### 3.3.3 TIM‐3 Inhibitors in Combination With PD‐1/PD‐L1 Inhibitors

TIM‐3 is highly expressed on exhausted T cells, Tregs, NK cells, and myeloid cells such as macrophages and MDSCs within the TME [67, 68, 69]. Notably, increased TIM‐3 expression on tumor‐infiltrating lymphocytes (TILs) and other immune cells in GC often correlates with poorer prognosis [70, 71]. The rationale for co‐targeting TIM‐3 and PD‐1 in GC stems from their frequent co‐expression on exhausted tumor‐infiltrating T cells. Since these pathways independently contribute to T cell dysfunction and PD‐1 inhibition can upregulate TIM‐3, combined targeting may more effectively restore T cell activity and overcome resistance [69].

Given the limited efficacy of anti‐TIM‐3 monotherapy, the primary focus of clinical development has been on combining TIM‐3 inhibitors with PD‐1/PD‐L1 blockade [72]. Several TIM‐3 inhibitors, such as TQB2618, bc3402, and cobolimab, are undergoing clinical trials in combination with PD‐1/PD‐L1 inhibitors for the treatment of advanced squamous cell carcinoma of the head and neck, liver cancer, non‐small cell lung cancer, and melanoma, with studies progressing at various stages. However, clinical evidence for TIM‐3‐targeted therapies specifically in GC still remains limited.

#### 3.3.4 TIGIT Inhibitors in Combination With PD‐1/PD‐L1 Inhibitors

T‐cell immunoreceptor with Ig and ITIM domains (TIGIT) is another key immune checkpoint receptor upregulated in various malignancies, including GC. Increased TIGIT expression on CD8^+^ T cells is associated with reduced cytokine production, increased apoptosis, diminished proliferative and cytotoxic capacity, and ultimately, poorer prognosis [73]. The rationale for co‐targeting TIGIT and PD‐1 in GC is strengthened by their synergistic interactions, which go beyond the commonly observed PD‐1 blockade‐induced TIGIT upregulation [74]. Crucially, both TIGIT and PD‐1 signaling pathways converge to suppress the vital co‐stimulatory receptor CD226. TIGIT achieves this mainly by outcompeting CD226 for their shared ligand CD155, while PD‐1 activation can indirectly lead to CD226 dephosphorylation [11, 75]. Crucially, because these pathways impose separate inhibitory brakes on CD226, neither TIGIT nor PD‐1 blockade alone is sufficient to fully restore CD226 function [76]. Therefore, dual blockade of TIGIT and PD‐1 is hypothesized to more effectively restore CD226‐mediated co‐stimulation, potentially reversing T cell exhaustion and enhancing anti‐tumor immunity.

Results from a phase II trial evaluating the TIGIT monoclonal antibody domvanalimab with zimberelimab (PD‐1 inhibitor) and FOLFOX (folinic acid, fluorouracil, oxaliplatin) in advanced gastroesophageal tumors were reported at the 2024 ESMO (European Society for Medical Oncology) Congress. As of March 2024, the study demonstrated an ORR of 59% and a mPFS of 12.9 months, indicating promising efficacy. Notably, patients with high PD‐L1 expression showed even greater benefits, with an ORR of 69% and an mPFS of 13.8 months [77]. Beyond combinations of separate monoclonal antibodies, innovative approaches such as bispecific antibodies are also being explored. For instance, a bispecific antibody targeting both PD‐1 and TIGIT is currently being assessed in a phase II clinical trial for its efficacy and safety in the perioperative treatment of HER2‐positive, unresectable locally advanced GC in combination with trastuzumab and chemotherapy (NCT06630130).

#### 3.3.5 LILRB1 Inhibitors in Combination With PD‐1/PD‐L1 Inhibitors

LILRB1, expressed on NK cells, macrophages, and cytotoxic lymphocytes, is an immunomodulatory receptor [78, 79]. It functions as an inhibitory receptor for HLA‐I molecules; upon binding, it diminishes the activation and cytotoxicity of LILRB1‐expressing NK cells and CD8^+^ T cells, thereby suppressing both innate and adaptive immune responses [80, 81]. Given its role in tumor immunity, LILRB1 is considered an atypical “immune checkpoint”. The HLA‐I/LILRB1 pathway is often overactivated in the TME, contributing to tumor immune evasion [82, 83]. In the GC microenvironment, LILRB1‐positive tumor‐associated macrophages (TAMs) typically exhibit an M2‐like phenotype. Furthermore, LILRB1 expression is associated with increased levels of immune checkpoints such as PD‐1 and CTLA‐4, elevated levels of inhibitory cytokines, and T‐cell exhaustion [84]. Preclinical studies have demonstrated that blocking the HLA‐I/LILRB1 signaling pathway can enhance the antitumor functions of macrophages and promote the activation of NK cells and cytotoxic T lymphocytes, potentially synergizing with ICIs [85]. Although clinical data confirming the role of LILRB1‐targeted therapy are lacking, several inhibitors are in Phase I/II trials with PD‐1 inhibitors for solid tumors [86].

Dual immune checkpoint blockade represents a promising strategy to overcome the limitations of PD‐1/PD‐L1 monotherapy in GC. Co‐targeting alternative checkpoints such as CTLA‐4, LAG‐3, TIGIT, or LILRB1 may enhance T and NK cell activation, reverse immunosuppressive phenotypes, and improve response durability. However, these benefits must be weighed against an increased risk of immune‐related adverse events. Future research should focus on identifying predictive biomarkers to guide patient selection, optimizing combination regimens, and balancing efficacy with tolerability.

### 3.4 VEGF/VEGFR Inhibitors in Combination With PD‐1/PD‐L1 Inhibitors

Activation of the vascular endothelial growth factor (VEGF) signaling pathway, a critical proangiogenic factor in tumor growth and metastasis, not only promotes tumor angiogenesis but also exerts immunosuppressive effects within the TME [87]. Specifically, VEGF pathway activation can increase the expression of inhibitory receptors such as PD‐1, leading to cytotoxic T lymphocyte (CTL) exhaustion and inducing immunosuppression [88]. Research by Gabrilovich et al. demonstrated that VEGF released by tumor cells can impair the maturation of precursor DCs [89]. Moreover, elevated VEGF levels recruit immunosuppressive cells such as Tregs and MDSCs, promoting their proliferation [90, 91]. Anti‐VEGF therapy can alleviate the VEGF‐mediated suppression of T cells, normalize the tumor vasculature, enhance immune cell infiltration, and synergistically improve the efficacy of PD‐1/PD‐L1 inhibitors [92].

Currently, treatment strategies that combine VEGF inhibitors with PD‐1/PD‐L1 inhibitors have shown significant efficacy across various cancer types [93, 94, 95]. The phase II EPOC1706 trial demonstrated encouraging antitumor activity for the combination of lenvatinib and pembrolizumab in previously untreated or refractory advanced GC/GEJC, reporting an ORR of 69% [96]. However, results from the subsequent phase III LEAP‐015 trial, presented at the 2025 ASCO Annual Meeting, were less favorable. In patients with HER2‐negative, PD‐L1 CPS ≥ 1 gastric cancer, the addition of lenvatinib and pembrolizumab to chemotherapy failed to significantly improve OS compared to chemotherapy alone (12.6 vs. 12.9 months) [97]. These findings suggest that while anti‐VEGFR and PD‐1/PD‐L1 combinations hold potential, their clinical benefit may be restricted to select patient subgroups. Our team is conducting two phase II clinical trials to evaluate the efficacy and safety of sintilimab in combination with the monoclonal antibody bevacizumab biosimilar IBI305 and chemotherapy, either as a first‐line treatment (NCT05640609) or as neoadjuvant therapy (NCT06667050) for advanced or locally advanced GC/GEJC. Unlike tyrosine kinase inhibitors, monoclonal antibodies offer more selective VEGF pathway blockade, potentially resulting in different immune‐modulatory profiles. Trial data are currently pending.

In parallel, novel dual‐targeting approaches are being explored. AK112 is a PD‐1/VEGF bispecific antibody that has demonstrated promising results in clinical studies for lung cancer treatment [98]. AK112 offers potential advantages over combining separate agents by simultaneously targeting both pathways, potentially simplifying treatment and enhancing synergistic effects. Currently, a clinical trial is evaluating the efficacy of AK112 in combination with AK104 and chemotherapy in patients with HER2‐negative advanced GC/GEJC (NCT06196697).

In summary, while anti‐VEGF/VEGFR therapies combined with PD‐1/PD‐L1 inhibitors are generally well tolerated and hypertension is the most frequently reported grade ≥ 3 adverse event, results from phase III trials have not met expectations. These findings highlight the need to more precisely identify patient subgroups most likely to benefit, such as those with AFP‐producing GC, and to develop robust predictive biomarkers through further analysis of existing data.

### 3.5 HER‐2‐Targeted Therapy in Combination With PD‐1/PD‐L1 Inhibitors

The combination of HER2‐targeted therapy with PD‐1/PD‐L1 inhibitors has demonstrated synergistic effects in cancer treatment, primarily due to their combined modulation of the immune microenvironment and tumor cells. Preclinical studies indicate that anti‐HER2 therapy can upregulate tumor cell PD‐L1 expression, for instance, via the interferon‐γ pathway, potentially representing an adaptive immune evasion mechanism. This upregulation might represent a tumor's adaptive response to evade immune attacks. When combined with PD‐1 inhibitors, it can effectively block the PD‐1/PD‐L1 pathway's suppression of T‐cell activity, thereby amplifying the anti‐tumor immune response [99]. However, the mechanisms linking HER2 signaling to PD‐L1 expression are complex, with other pathways like PI3K‐AKT–mTOR also implicated [100]. Additionally, trastuzumab may enhance DC presentation of HER2 antigens via its Fc region, promoting HER2‐specific T cell infiltration and a coordinated immune response [101]. Although the precise molecular mechanisms remain incompletely understood, the synergy between anti‐HER2 therapy and ICIs has been further validated in multiple clinical studies on GC.

Combining trastuzumab, which targets HER2 via cytotoxic mechanisms, with PD‐1/PD‐L1 inhibitors that enhance adaptive immunity can augment antitumor efficacy. The KEYNOTE‐811 study evaluated the combination of pembrolizumab and trastuzumab as first‐line therapy in HER2‐positive advanced GC/GEJC patients. The final analysis demonstrated that the pembrolizumab combination significantly improved mOS (20.0 vs. 16.8 months) and mPFS (10.0 vs. 8.1 months). Patients with PD‐L1 CPS ≥ 1 experienced a more pronounced mOS benefit (20.1 vs. 15.7 months). The ORR was also higher with pembrolizumab (72.6% vs. 60.1%). Grade ≥ 3 adverse events were slightly more frequent in the pembrolizumab arm (59% vs. 51%). These findings establish this combination as a new standard of care for first‐line HER2‐positive advanced GC/GEJC, especially for patients with PD‐L1 CPS ≥ 1 [102].

Zanidatamab is a bispecific antibody targeting HER2, demonstrating stronger ADCC and CDC activity compared to HER2 monoclonal antibodies. Based on prior study results, Zanidatamab has been approved by the FDA for the treatment of GC. A Phase Ib/II clinical study is evaluating the efficacy of zanidatamab combined with CAPOX chemotherapy and tislelizumab as a first‐line treatment for advanced HER2‐positive GC/GEJC. According to data presented at the 2023 ESMO conference, the ORR reached 75.8%, with a mPFS of 16.7 months [103]. These promising results support further investigation in the ongoing phase III HERIZON‐GEA‐01 trial (NCT05152147). In addition, clinical trials evaluating HER2‐targeting antibody‐drug conjugates (ADCs) combined with immunotherapy are also underway.

### 3.6 FGFR2‐Targeted Therapy in Combination With PD‐1/PD‐L1 Inhibitors

Fibroblast growth factor receptor 2 (FGFR2) expression is often dysregulated in tumors through gene amplification, point mutations, or chromosomal rearrangements. Studies have shown that overexpression of FGFR2 is associated with reduced T‐cell infiltration in tumors, leading to resistance to PD‐1 inhibitors in such patients [104]. FGFR2 gene amplification or protein overexpression correlates with poor prognosis in GC [105]. Blocking FGFR2 can inhibit downstream signaling pathways, such as MAPK, PI3K/AKT, and JAK/STAT, resulting in enhanced CD8^+^ T‐cell infiltration, thereby improving the efficacy of PD‐1 inhibitors [104]. Despite this promising preclinical rationale, robust clinical evidence supporting the combination of FGFR2 inhibitors with PD‐1/PD‐L1 inhibitors in GC is still nascent. Currently, a Phase II clinical trial is evaluating the combination of futibatinib (an anti‐FGFR2 agent) and pembrolizumab with chemotherapy in patients with unresectable or metastatic solid tumors, including Siewert Type I gastroesophageal junction cancer (NCT05945823).

### 3.7 DKK1‐Targeted Therapy in Combination With PD‐1/PD‐L1 Inhibitors

Dickkopf‐related protein 1 (DKK1) is a secreted protein that inhibits the Wnt/β‐catenin signaling pathway by binding to the Wnt co‐receptors LRP5/6. Additionally, DKK1 can indirectly modulate the PI3K/AKT pathway by enhancing the activity of immunosuppressive M2 macrophages within the TME, thus contributing to tumor progression and immune evasion [106]. High DKK1 expression in GC correlates with tumor progression, poor prognosis, and an immunosuppressive TME characterized by increased M2 macrophages and reduced antitumor CD4^+^ T and NK cells [106, 107, 108, 109]. DKN‐01, a monoclonal antibody targeting DKK1, can neutralize DKK1, reprogramming M2 macrophages into the antitumor M1 phenotype and increasing the infiltration of immune effector cells such as CD8^+^ T cells, thereby enhancing the antitumor immune response [110]. Crucially, this DKK1 blockade‐mediated reprogramming of TAMs has been shown in preclinical GC models to restore immune activity within the TME and augment the efficacy of PD‐1 Inhibitors [109].

Recently, a Phase II clinical study evaluated the combination of DKN‐01 with tislelizumab (a PD‐1 monoclonal antibody) and chemotherapy as a first‐line treatment for advanced GC/GEJC, yielding promising results. The ORR reached 90% in patients with high DKK1 expression, and even in patients with low PD‐L1 expression, the ORR remained as high as 86% [111]. Importantly, the combination regimen demonstrated a manageable safety profile, with the most common DKN‐01‐related adverse events being gastrointestinal in nature and generally low‐grade. This strategy addresses the current gap in effective targets for GC treatment and offers a novel approach for immunosensitization, potentially overcoming immune resistance.

### 3.8 ADCs in Combination With PD‐1/PD‐L1 Inhibitors

ADCs are innovative agents combining monoclonal antibody specificity with potent cytotoxic payloads, enabling targeted delivery to antigen‐expressing tumor cells to maximize killing while minimizing systemic toxicity [112]. Preclinically, ADCs can promote ICD by releasing cytotoxic compounds, a mechanism of immunosensitization shared with therapies like chemotherapy and radiotherapy, thus stimulating antitumor immunity [113]. A key feature of ADCs is the bystander effect, where released payloads kill nearby antigen‐negative tumor cells, enhancing efficacy in heterogeneous tumors. HER2, claudin18.2, and Trop2 are frequently overexpressed in GC and have low or absent expression in normal tissues, making them ideal targets for ADCs [114, 115]. In recent years, ADCs like RC48 and trastuzumab deruxtecan (T‐DXd, DS‐8201) have seen success in treating GC and are now approved for clinical use. However, the efficacy of combining ADCs with PD‐1/PD‐L1 inhibitors still requires validation through larger clinical studies, with ongoing trials evaluating ADCs targeting HER2, claudin18.2, or Trop2 in combination with PD‐1 inhibitors for GC treatment.

HER2‐targeting ADCs are currently the most advanced, exemplified by T‐DXd and RC48. Preliminary results from the DESTINY‐Gastric03 trial demonstrated promising efficacy of first‐line treatment combining T‐DXd, fluoropyrimidine‐based chemotherapy, and pembrolizumab. But tolerability was suboptimal at the 6.4 mg/kg T‐DXd dose. Specifically, treatment‐related interstitial lung disease (ILD) was reported in 8 of 43 patients in this cohort, including 2 fatal cases [116]. Consequently, the ongoing phase III DESTINY‐Gastric05 trial is evaluating a reduced T‐DXd dose of 5.4 mg/kg to further explore its efficacy and safety in the first‐line setting. Similarly, RC48 combined with PD‐1 inhibitors has shown encouraging clinical outcomes. Recent data from the phase II RCTS trial reported an impressive ORR of 89.4% and a median PFS of 12.7 months with RC48 plus tislelizumab and S‐1 chemotherapy as first‐line therapy in patients with advanced GC (HER2 IHC ≥ 2+). This benefit was even more pronounced in the HER2‐positive or PD‐L1 ≥ 1% patient subgroups, underscoring the regimen's significant potential [117]. Currently, a phase III trial (NCT06944496) is ongoing to evaluate the efficacy and safety of RC48 combined with standard chemotherapy in first‐line therapy for advanced gastric cancer with low HER2 expression. In contrast, clinical data on ADCs targeting CLDN18.2 or Trop2 combined with PD‐1/PD‐L1 inhibitors have not yet been reported, although relevant trials are actively underway (Table 2). Overall, combining ADCs with PD‐1/PD‐L1 inhibitors holds substantial promise, but overlapping toxicities, particularly interstitial pneumonitis, may significantly elevate the incidence of adverse events. Therefore, further evaluation and optimization of safety and clinical feasibility are critical to advancing these combination regimens.

**TABLE 2:** Clinical Trials of PD‐1/PD‐L1 Inhibitor‐Based Combination Therapies in GC/GEJC.

Year	Study ID/Name	Trial phase	Patients Enrolled	PD‐1/PD‐L1 inhibitor	Combination strategy	Lines	Patient population	Prmary outcome (month)	References
2022	NCT03448835 (PANDA)	2	20	Atezolizumab	XOD	Neoadjuvant	Non‐metastatic GC/GEJC, Resectable	pCR 45% MPR 70%	[118]
2022	NCT03631615 (Neo‐PLANET)	2	36	Camrelizumab	XELOX + CFRT	Neoadjuvant	Locally advanced GC, Resectable	pCR 33.3% MPR 44.4% R0 91.7%	[119]
2022	NCT04006262 (GERCOR NEONIPIGA)	2	32	Nivolumab	Ipilimumab (anti‐CTLA4 Ab)	Neoadjuvant	MSI/dMMR Locally advanced GC/GEJC, Resectable	pCR 58.6%	[54]
2023	CTR1900024428	2	34	Sintilimab	SOX + CFRT	Neoadjuvant	Locally advanced GC, Resectable	pCR 18.3% mDFS 17.0% mEFS 21.1%	[120]
2023	NCT03064490 (PROCEED)	2	35	Pembrolizumab	Metronomic Carbo‐Pac + CFRT	Neoadjuvant	Locally advanced, GC/GEJC, Resectable	pCR 35.7%	[40]
2023	NCT04817826 (INFINITY)	2	18	Durvalumab	Tremellmumab (anti‐CTLA4 Ab)	Neoadjuvant	MSI/dMMR Locally advanced GAC/GEJAC, Resectable	pCR 60%	[55]
2024	NCT03878472	2	30	Camrelizumab	Apatinib (VEGFR2‐TKI) + SOX	Neoadjuvant	Locally advanced GC, Resectable	pCR 15.8% MPR 26.3% R0 82.6%	[121]
2017	NCT03257163	2	Planned: 40	Pembrolizumab	Capecitabine + Radiation Therapy	Neoadjuvant	dMMR+ and EB + GC, Resectable	RFS (On going)	—
2023	NCT06054906	2	Planned: 50	Sintilimab	Metronomic PLOF	Neoadjuvant	Locally advanced GC. Resectable	pCR, MPR (On going)	—
2024	NCT06667050	2	Planned: 58	Sintilimab	IBI305 (anti‐VEGF Ab) + XELOX	Neoadjuvant	Locally advanced GC, Resectable	pCR (On going)	—
2024	NCT06426654	1/2	Planned: 45	Sintilimab	LDRT	Neoadjuvant	dMMR/MSI‐H, Locally advanced GC/GEJC. Resectable	pCR (On going)	—
2024	NCT06266871	1/2	Planned: 64	Tiselizumab	LDRT + SOX	Neoadjuvant	Localized/locally advanced GC/GEJC, Resectable	pCR (On going)	—
2020	NCT02494583 (KEYNOTE‐062)	3	257	Pebrolzumab	FP/XP	1	PD‐L1 CPS ≥ 1, HER2‐, advanced/metastatic GC/GEJC, Unresectable	mOS 12.5 mPFS 6.9	[122]
2023	NCT03675737 (KEYNOTE‐859)	3	790	Pebrolzumab	FP/XELOX	1	HER2‐, advanced/metastatic GC/GEJC, Unresectable	mOS 12.9	[24]
2021	NCT02872116 (CHECKMATE‐649)	3	789	Nivolumab	XELOX/FOLFOX	1	PD‐L1 CPS ≥ 5, HER2‐, advanced/metastatic GC/GEJC/EAC, Unresectable	mOS 14.4 mPFS 7.7	[25]
2025	NCT04662710 (LEAP‐015)	3	443	Pembrolizumab	Lenvatinib (Multi‐target TKI) + CAPOX/FOLFOX	1	HER2‐, Locally advanced/metastatic GC/GEJC, Unresectable	(PD‐L1 CPS ≥ 1) ORR 59.5% mOS 7.3 mPFS 12.6	[97]
2023	NCT03745170 (ORIENT‐16)	3	327	Sintilimab	XELOX	1	HER2‐, advanced/metastatic GC/GEJC, Unresectable	mOS 11.7 mPFS 7.0	[26]
2023	NCT03615326 (KEYNOTE‐811)	3	350	Pembrolizumab	Trastuzumab (anti‐HER2 Ab) + FP/CAPOX	1	HER2+, Locally advanced/metastatic GC/GEJC, Unresectable	mOS 20.0 mPFS 10.0	[102]
2024	NCT05008783 (COMPASSION‐15)	3	305	Cadonilimab	XELOX	1	HER2‐, advanced/metastatic GC/GEJC, Unresectable	ORR 65.2% mOS 14.1 mPFS 7.0	[57]
2023	NCT05329766 (EDGE‐Gastric)	2	41	Zimberelimab	Domvanalimab (anti‐TIGIT Ab) + FOLFOX	1	Arm A1: HER2‐, advanced/metastatic GC/GEJC/EAC, Unresectable	ORR 59%	[77]
2024	NCT03662659 (RELATIVITY‐060)	2	136	Nivolumab	Relatlimab (anti‐LAG3 Ab) + FOLFOX	1	HER2‐, advanced/metastatic GC/GEJC, Unresectable	ORR 48% mOS 13.5 mPFS 7.0	[66]
2024	NCT04363801	2	25	Tiselizumab	DKN‐01 (anti‐DKK1 Ab) + XELOX	1	Part A: Locally advanced/metastatic GC/GEJC, Unresectable	ORR 73%	[111]
2024	NCT04757363	2	39	Nivolumab	Regorafenib (Multi‐target TKI) + FOLFOX	1	HER2‐, Locally advanced/metastatic GC/GEJC, Unresectable	mPFS 13.0	[123]
2025	NCT04609176	2	36	Camrelizumab	Apatinib + SOX	1	HER2‐, AFP > 2UIN/IHC AFP+, advanced/metastatic GC/GEJC, Unresectable	ORR 66.7% DCR 88.9% mOS 18.0 mPFS 7.8	[124]
2024	NCT05586061 (RCTS)	2	57	Tislelizumab	RC48 (HER2‐ADC) + S‐1	1	HER2 IHC ≥ 2 advanced/metastatic GC/GEJC, Unresectable	ORR 89.4% mPFS 12.7	[117]
2022	NCT04276493	1/2	33	Tiselizumab	Zanidatamab (HER‐2 BsAb) + CAPOX	1	Arm‐2: HER2+, locally advanced/metastatic GC/GEJC, Unresectable	ORR 72.7% mPFS 10.9	[103]
2021	NCT05144854	3	Planned: 300	Nivolumab	Ipilimumab + FP/XELOX	1	HER2‐ advanced/recurrent GC, Unresectable	OS (On going)	—
2021	NCT05008783	3	Planned: 250	Cadonilimab (anti–PD‐1/CTLA‐4 BsAb)	XELOX	1	Locally advanced/metastatic GC/GEJC, Unresectable	OS (On going)	—
2021	NCT05152147 (HERIZON‐GEA‐01)	3	Planned: 357	Tislelizumab	Zanidatamab (anti‐HER2 BsAb) + CAPOX/FP	1	HER2+ advanced/metastatic GC/GEJC, Unresectable	PFS, OS (On going)	—
2025	NCT06731478 (DESTINY Gastric05)	3	Planned: 363	Pembrolizumab	T‐DXd (HER2‐ADC) + Capecitabine/5‐FU	1	HER2+ advanced/metastatic GC/GEJC, Unresectable	PFS (On going)	—
2025	NCT06944496	3	Planned: 308	Tislelizumab	RC48 + CAPOX	1	HER2‐low advanced/metastatic GC/GEJC, Unresectable	PFS (On going)	—
2021	NCT05007106 (KEYVIBE‐005)	2	Planned: 480	Pembrolizumab	Vibostolimab (anti‐TIGIT Ab)	1	PD‐L1 CPS ≥ 1, Advanced/metastatic Solid tumors, Unresectable	ORR, PFS (On going)	—
2023	NCT06093425	3	Planned: 475	Nivolumab	Osemitamab (anti‐CLDN18.2 Ab) + FOLFOX/CAPOX	1	Claudin18.2 +, HER2‐, Locally advanced/metastatic GC/GEJC, Unresectable	PFS (On going)	—
2023	NCT05945823	2	Planned: 13	Pembrolizumab	Futibatinib (FGFR‐TKI) + FP/FOLFOX	1	Arm A: HER2‐, Locally advanced EC/GEJC, Unresectable	ORR (On going)	—
2022	NCT05568095	3	Planned: 520	Zimberelimab	Domvanalimab (anti‐PD‐1 Ab) + FOLFOX/CAPOX	1	Locally advanced/metastatic GC/GEJC, Unresectable	OS (On going)	—
2023	NCT05640609	1/2	Planned: 57	Sintilimab	Bevacizumab + CAPOX	1	HER2‐, advanced/metastatic GC/GEJC	DLT, ORR (On going)	—
2024	NCT06196697	2	Planned: 50	Cadonilimab and AK102	SOX/XELOX	1	HER2‐, Locally advanced/metastatic GC/GEJC, Unresectable	ORR (On going)	—
2024	CTR2400089068	1	Planned: 18	Tiselizumab	LDRT + SBRT + CAPOX	1	HER2‐ advanced/recurrent GC, Unresectable	Safety, Tolerability (On going)	—
2024	NCT06586294	1/2	Planned: 147	AK129 (anti PD‐1/LAG‐3 BsAb) + Cadonilimab	CAPOX	1	HER2‐, Locally advanced/metastatic GC/GEJC, Unresectable	AE, DLT, ORR (On going)	—
2024	NCT06742411	1/2	Planned: 57	Sintilimab	HBOT + XELOX	1	HER2‐, advanced/metastatic GC/GEJC	ORR (On going)	—
2020	NCT03609359 (EPOC1706)	2	29	Pembrolizumab	Lenvatinib	1/2	Metastatic/recurrent GC/GEJC	ORR 69%	[96]
2022	NCT04713059	2	62	Toripalimab	Anlotinib	2	Advanced/metastatic GC/GEJC, Unresectable	mOS 11.1 mPFS 4	[125]
2021	UMIN000025947	1/2	43	Nivolumab	Ramucirumab (anti–VEGFR2 Ab) + Paclitaxel	2	Advanced/recurrent GC/GEJC, Unresectable	mOS 13.1 mPFS 5.1	[126]
2024	NCT06341335	3	Planned: 253	Cadonilimab	Paclitaxel	2	HER2‐, advanced/metastatic GC/GEJC, Unresectable	PFS, OS (On going)	—
2020	NCT04385550	3	Planned: 264	AK105	Anlotinib (Multi‐target TKI)	2	Locally advanced/metastatic GC/GEJC, Unresectable	OS (On going)	—
2020	NCT04209686	2	Planned: 36	Pembrolizumab	Olaparib (PARP inhibitor) + Paclitaxel	2	Advanced GC	OS (On going)	—
2023	NCT05620628 (VIKTORY‐2)	2	Planned: 25	Durvalumab	Savolitinib (MET‐TKI)	2	Advanced MET Amplified GC/GEJC	PFS (On going)	—
2024	NCT06321913	2	Planned: 25	Sintilimab	IBI343 (TROP2‐ADC)	2	Locally advanced/metastatic GC/GEJC, Unresectable	ORR, AE (On going)	—
2024	NCT06251973	2	Planned: 37	Balstilimab	agenT‐797 (Invariant Natural Killer T Cells) + Botensilimab (anti‐CTLA4 Ab) + Ramucirumab+Paclitaxel	2	Advanced/metastatic GC/GEJC/EAC, Unresectable	ORR (On going)	—
2020	NCT02572687 (JVDJ)	1	29	Durvalumab	Ramucirumab	3	GC/GEJC cohort: Locally advanced/metastatic GC/GEJC, Unresectable	ORR 21% mOS 12.4 mPFS 2.6	[127]
2024	NCT03453164	1/2	41	Nivolumab	SBRT	3	Advanced/recurrent GC, Unresectable	DCR 22.5%	[128]
2024	NCT04280341	1	30	Toripalimab	RC48	> 1	HER2 IHC ≥ 1/ISH+, advanced solid tumors (GC/GEJC Arm)	ORR 43% mOS 16.8 mPFS 6.2	[129]
2022	NCT05188664	1/2	Planned: 50	Toripalimab	LM‐302 (CLDN18.2‐ADC)	> 1	Advanced solid tumors (no separate GC/GEJC cohort specified)	MTD, OBD (On going)	—
2023	NCT05941507	1/2	Planned: 300	anti‐PD‐1 Ab	LCB84 (TROP2‐ADC)	> 1	Advanced solid tumors (no separate GC/GEJC cohort specified)	MTD, RP2D, AE, PFS, OS, ORR (On going)	—
2023	NCT06088004	1/2	Planned: 218	Toripalimab	ABO2011 (CLDN18.2‐ADC)	> 1	Advanced Solid Tumor (no separate GC/GEJC cohort specified)	DLT, SAE, ORR (On going)	—
2023	NCT05763004	1	Planned: 140	Pembrolizumab	IOS 1002 (LILRB antagonist)	> 1	Advanced solid tumors (no separate GC/GEJC cohort specified)	AE (On going)	—
2023	NCT05379972	2	Planned: 26	Pembrolizumab	SBRT+Olaparib	≥ 2	Homologous recombination deficiency, metastatic GC/GEJC	ORR (On going)	—
2021	NCT04879368	3	Planned: 225	Nivolumab	Regorafenib	> 2	Locally advanced/metastatic GC/GEJC, Unresectable	OS (On going)	—
2022	NCT05163483	2	Planned: 87	Toripalimab	Tucidinostat (HDAC inhibitor) + Bevacizumab	≤ 4	Advanced/metastatic GC/GEJC/EAC, Unresectable	ORR (On going)	—
2024	NCT06630130	2	Planned: 25	Rilvegostomig (anti–PD‐1/LAG‐3 BsAb)	T‐DXd + Capecitabine	—	HER2+, Locally advanced GC/GEJC, Unresectable	AE (On going)	—
2021	NCT04913337	1/2	Planned: 179	Pembrolizumab	NGM707 (anti‐LILRB1/LILRB2 BsAb)	—	Advanced Solid Tumor (no separate GC/GEJC cohort specified)	DLT, ORR, OS (On going)	—
2022	NCT05377528	1	Planned: 22	Balstilimab	AGEN1571 (anti‐LILRB1 Ab) + Botensilimab	—	Advanced solid tumors (no separate GC/GEJC cohort specified)	AE (On going)	—

*Note:* ‘Patients Enrolled’ indicates the cohort size for the specific combination strategy listed, or total enrollment for single‐arm trials.

Abbreviations: AE, Adverse Event; CAPOX, Capecitabine and Oxaliplatin; Carbo‐Pac, Carboplatin and Paclitaxel; DCR, Disease Control Rate; DFS, Disease‐Free Survival; DLT, Dose‐Limiting Toxicity; DOR, Duration of Response; EFS, Event‐Free Survival; FOLFOX, Folinic acid, Fluorouracil, and Oxaliplatin; FP, 5‐Fluorouracil Cisplatin; mDFS, median Disease‐Free Survival; mDOR, median Duration of Response; mEFS, median Event‐Free Survival; mOS, median Overall Survival; mPFS, median Progression‐Free Survival; MPR, Major Pathological Response; ORR, Objective Response Rate; OS, Overall Survival; PCR/pCR, Pathological Complete Response; PFS, Progression‐Free Survival; PLOF, Paclitaxel, Oxaliplatin and 5‐Fluorouracil; R0, No residual tumor (microscopic); RFS, Relapse‐Free Survival; SOX, S‐1 and Oxaliplatin; TRAE, Treatment‐Related Adverse Event; XELOX, Capecitabine and Oxaliplatin; XOD, Capecitabine, Oxaliplatin, and Docetaxel; XP, Capecitabine, Cisplatin.

### 3.9 Epigenetic and DNA Repair Regulators in Combination With PD‐1/PD‐L1 Inhibitors

#### 3.9.1 PARP Inhibitors in Combination With PD‐1/PD‐L1 Inhibitors

Poly (ADP‐ribose) polymerase (PARP) inhibitors exert their therapeutic effects by inhibiting DNA repair, leading to DNA double‐strand breaks and irreparable damage in tumor cells with homologous recombination deficiencies (HRDs) or BRCA mutations [130]. This disruption significantly increases the TMB and promotes the release of neoantigens, thereby enhancing the immunogenicity of the tumor. In the immune system, PARP inhibitor‐induced DNA damage can activate the cGAS‐STING pathway, triggering the production of type I interferons and the upregulation of chemokines such as CCL5 and CXCL10. These factors attract and activate CD8^+^ T cells in the TME, creating a more favorable setting for immune‐mediated tumor elimination [131].

PARP inhibitor‐induced DNA damage can lead to an adaptive upregulation of PD‐L1 on tumor cells, which, while potentially a resistance mechanism, can be effectively counteracted by concurrent PD‐1/PD‐L1 blockade, thereby restoring T‐cell function and boosting antitumor immunity [132]. Additionally, PARP inhibition can trigger TH1‐type immune responses and inflammatory signaling in the TME, further enhancing tumor immunogenicity and ICI responsiveness [133].

The MEDIOLA study provided preliminary evidence for combining PARP and PD‐1 inhibitors in breast and ovarian cancers [134]. However, the 2024 ESMO data from the ATHENA‐COMBO trial indicated that for ovarian cancer patients, the combination of the PARP inhibitor rucaparib and the PD‐1 inhibitor nivolumab showed inferior efficacy compared with rucaparib monotherapy, likely due to treatment discontinuation caused by severe adverse events [135]. In GC, while several trials are evaluating this combination, published data from completed studies are limited, and its therapeutic value remains to be established.

#### 3.9.2 LSD1 Inhibitors in Combination With PD‐1/PD‐L1 Inhibitors

Lysine‐specific demethylase 1 (LSD1) regulates gene expression by demethylating H3K4me1/2 and H3K9me1/2 [136]. Preclinical studies have shown that LSD1 is overexpressed in GC and contributes to tumor growth and immune evasion through various mechanisms [137]. Additionally, LSD1 inhibits CD8^+^ T cell migration, infiltration, and cytotoxicity, as well as M1 macrophage polarization [138]. Recent studies show LSD1 suppresses T‐cell responses by promoting PD‐L1‐expressing exosome release; LSD1 knockout in GC cells reduced these exosomes and restored T‐cell responses [139, 140]. These findings suggest that LSD1 is a potential auxiliary target for PD‐1/PD‐L1 inhibitor therapy, though this combination remains preclinical.

#### 3.9.3 HDAC Inhibitors in Combination With PD‐1/PD‐L1 Inhibitors

The overexpression of histone deacetylases (HDACs) is commonly observed in various cancers, including GC. HDACs promote the deacetylation of both histone and non‐histone proteins, leading to functional impairments that contribute to tumor progression [141]. Effective T‐cell mediated tumor destruction relies on robust antigen presentation via MHC molecules, a process often subverted by cancer cells (e.g., through MHC downregulation) to evade immune attack. Preclinical studies have demonstrated that HDAC inhibitors can beneficially upregulate MHC molecule expression on tumor cells, enhancing antigen presentation, but these agents can also induce an adaptive upregulation of PD‐L1 expression, a potential mechanism of adaptive resistance. This dual effect strongly suggests that combining HDAC inhibitors with PD‐1/PD‐L1 blockade may be an optimal strategy to harness the pro‐immunogenic effects of HDAC inhibition while simultaneously neutralizing the upregulated PD‐L1 checkpoint [142]. Although triple combinations involving HDAC inhibitors, PD‐1 inhibitor, and antiangiogenic agents have shown promise in certain cancers, such strategies remain underexplored in GC [143].

**FIGURE 2:**
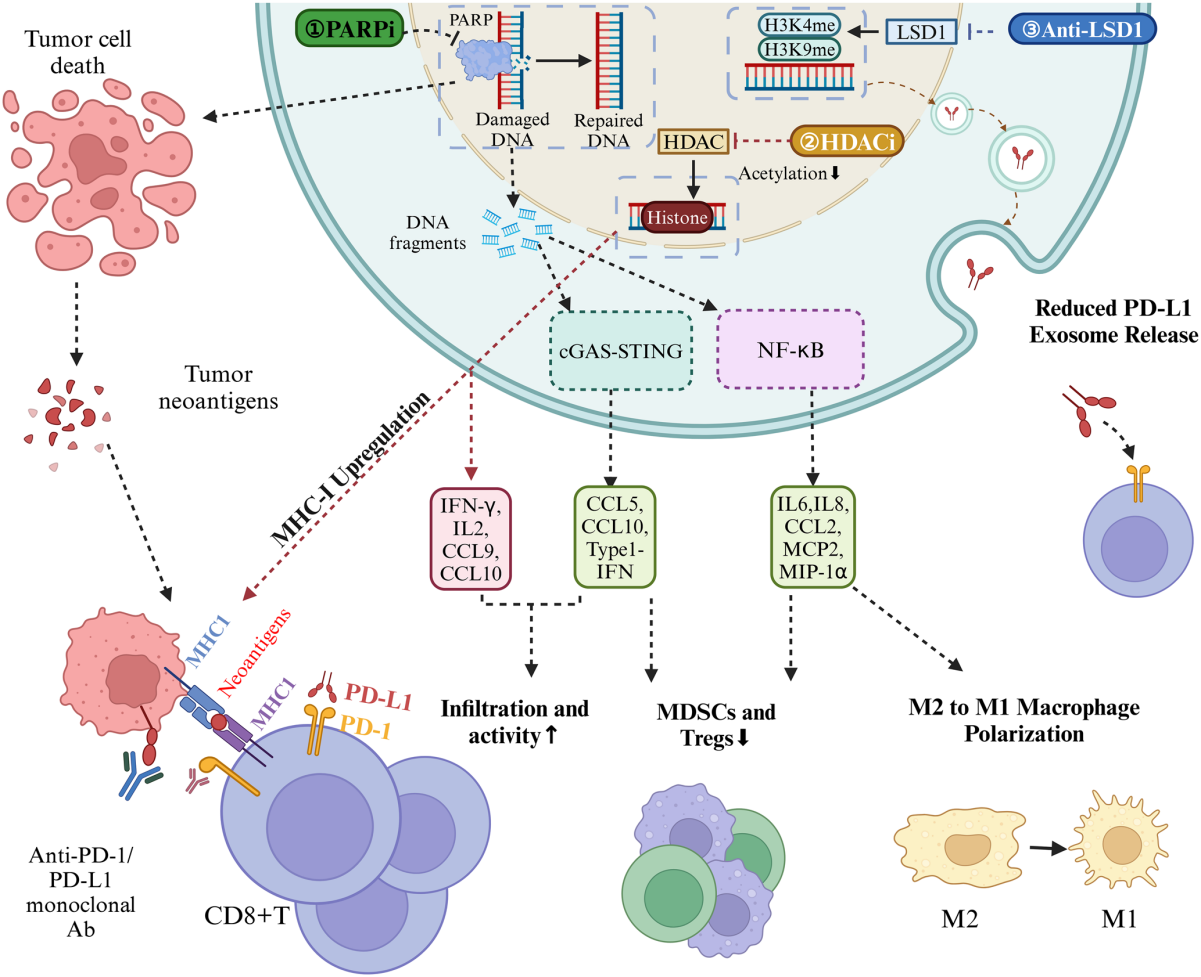
Mechanisms of epigenetic and DNA Repair regulators combined with PD‐1/PD‐L1 inhibitors in GC immunotherapy. This figure illustrates how epigenetic and DNA repair regulators potentiate PD‐1/PD‐L1 inhibitor efficacy in GC through distinct molecular mechanisms: (1) PARPi: PARPi induce DNA damage, leading to the accumulation and cytosolic leakage of DNA fragments in tumor cells. This activates the cGAS‐STING innate immune pathway, resulting in the release of type I interferons, CCL5, and CXCL10. Concurrently, NF‐κB activation triggers the secretion of pro‐inflammatory cytokines and chemokines (IL‐6, IL‐8, CCL2, MCP‐2, MIP‐1α). These events collectively promote an inflamed TME characterized by increased CD8^+^ T‐cell infiltration and activity, reduced MDSCs and Tregs, and polarization of macrophages from M2 to M1 phenotype. Additionally, PARPi can induce ICD, leading to the release of tumor neoantigens and enhanced antigen presentation. (2) HDACi: HDACi inhibit histone deacetylation, leading to a more open chromatin structure that facilitates the re‐expression of “silenced” immune‐related genes. This results in upregulation of MHC‐I molecules on tumor cells, enhancing antigen presentation. Furthermore, HDACi can promote the release of IFN‐γ, IL‐2, CCL9, and CXCL10, thereby boosting CD8^+^ T‐cell infiltration and activity. (3) Anti‐LSD1: LSD1 inhibitors act by inhibiting histone demethylation. A key immunomodulatory effect relevant to ICI synergy is their ability to reduce the release of PD‐L1‐containing exosomes from tumor cells. By diminishing this exosome‐mediated transfer of immunosuppressive PD‐L1 to other cells, LSD1 inhibitors can help restore T‐cell responsiveness and function. Created in https://BioRender.com. CCL2, Chemokine (C‐C motif) Ligand 2; CCL5, Chemokine (C‐C motif) Ligand 5; CCL9, Chemokine (C‐C motif) Ligand 9; cGAS‐STING, cyclic GMP‐AMP Synthase/Stimulator of Interferon Genes (pathway); CXCL10, Chemokine (C‐X‐C motif) Ligand 10; HDACi, Histone Deacetylase inhibitors; ICD, Immunogenic Cell Death; LSD1, Lysine‐Specific Demethylase 1; MCP‐2, Monocyte Chemoattractant Protein‐2; MIP‐1α, Macrophage Inflammatory Protein‐1 alpha; PARPi, Poly (ADP‐ribose) Polymerase inhibitors.

Epigenetic and DNA repair regulators, such as PARP, LSD1, and HDAC inhibitors, represent promising strategies for enhancing the efficacy of PD‐1/PD‐L1 inhibitors. These agents modulate the TME by increasing tumor immunogenicity, promoting antigen presentation, and restoring T‐cell functionality. Despite their potential, clinical evidence for these combination therapies in GC remains limited, underscoring the need for further research. The mechanisms underlying these combinations are illustrated in Figure 2.

### 3.10 Other Combination Therapies

#### 3.10.1 Fecal Microbiota Transplantation in Combination With PD‐1/PD‐L1 Inhibitors

In GC patients, the composition of the gut microbiota often undergoes significant changes, which are closely linked to tumor development, progression, and treatment response. GC patients often exhibit gut dysbiosis, characterized by reduced microbial diversity, an overgrowth of specific pathobionts like Prevotella species, and a depletion of beneficial bacteria such as Bifidobacterium and Lactobacillus [144]. This imbalance not only affects the patients' metabolic and immune status but may also promote tumor progression through inflammatory pathways [145].

In this context, modulating the gut microbiota, for instance through fecal microbiota transplantation (FMT), holds unique potential to enhance the efficacy of PD‐1/PD‐L1 inhibitors in GC treatment, given the crucial role of a diverse and healthy gut microbiome in immunotherapy responses [146]. Clinically, FMT from responders has shown promise in converting PD‐1 inhibitor non‐responders to responders in some melanoma patients, yielding significant clinical benefits [147].

More directly relevant to GC, Sunakawa et al. reported that the presence of specific bacteria like Odoribacter and Veillonella in the gut microbiota was associated with positive responses to nivolumab in GC patients [148]. However, current research on the role of the gut microbiota in GC immunotherapy remains limited, and further studies are needed to explore the potential relationship between the gut microbiome and PD‐1/PD‐L1 inhibitors in GC treatment.

#### 3.10.2 Autologous Cell Therapy in Combination With PD‐1/PD‐L1 Inhibitors

Autologous cell therapy (ACT) involves collecting a patient's T cells, expanding or genetically modifying them ex vivo before reinfusing this large pool of often tumor‐specific T cells to directly boost immune responses at the tumor site. Common approaches include TIL, CAR‐T, and TCR‐modified T‐cell therapies [149]. However, the efficacy of ACT is often limited by tumor immune evasion mechanisms, such as reduced MHC class I molecule expression and the upregulation of T‐cell immune checkpoints [150].

Given that PD‐1/PD‐L1 inhibitors can alleviate T‐cell exhaustion and restore antitumor activity, their combination with ACT offers a compelling synergistic strategy. These inhibitors can enhance the functionality and persistence of the reinfused therapeutic T cells by preventing their inactivation within the suppressive TME [151]. Although clinical data in GC are still limited, preliminary studies suggest that combining ACT with PD‐1 inhibitors can overcome immunosuppression in the TME, promoting T‐cell infiltration and function to improve antitumor efficacy [152]. Consequently, this combined approach may hold particular promise for GC patients with refractory disease or those who have exhausted conventional treatment options, potentially offering a novel avenue for achieving durable responses. Multiple trials are evaluating ACT combined with PD‐1 or CTLA‐4 inhibitors.

#### 3.10.3 Oncolytic Viruses in Combination With PD‐1/PD‐L1 Inhibitors

Oncolytic viruses (OVs) selectively infect and lyse tumor cells, inducing apoptosis and simultaneously promoting antitumor immune responses. Furthermore, OVs infection leads to the release of TAAs and danger signals, which activate innate immune cells like NK cells and DCs, as well as the adaptive immune system, thereby amplifying systemic antitumor immunity [153]. Combining OVs with PD‐1/PD‐L1 inhibitors can produce significant synergistic antitumor effects. While OVs can “heat up” the TME by destroying tumor cells and releasing antigens, PD‐L1 expression often increases, leading to T‐cell function inhibition [154].

Although this combination strategy has shown antitumor activity and survival benefits in glioblastoma [155], robust clinical data evaluating OVs combined with PD‐1/PD‐L1 inhibitors specifically in GC are currently scarce. Its potential to overcome TME immunosuppression and provide new hope for patients with refractory GC warrants further dedicated clinical trials.

#### 3.10.4 Co‐Stimulatory Receptor Agonists in Combination With PD‐1/PD‐L1 Inhibitors

Costimulatory receptors expressed on T cells, such as CD137 (4‐1BB) and CD27, can promote T‐cell proliferation and activation upon binding to their ligands, enhancing immune responses and increasing antitumour activity [156]. Targeting these receptors has emerged as a key strategy to improve immunotherapy efficacy. An innovative approach to simultaneously engage co‐stimulatory pathways and block inhibitory checkpoints involves the use of bispecific antibodies (BsAbs). Recently, BsAbs targeting both costimulatory receptors and PD‐1/PD‐L1 inhibitors have gained attention in cancer treatment. For example, MCLA‐145 is a BsAb that binds to both CD137 and PD‐L1, boosting antitumor effects by activating T cells and blocking PD‐L1‐mediated immunosuppression. It has shown stronger antitumor activity than anti‐PD‐1 antibodies alone in various tumor models [157]. In GC, where tumors often exhibit a highly immunosuppressive TME characterized by low PD‐L1 expression or poor T‐cell infiltration, rendering PD‐1/PD‐L1 inhibitors less effective, the addition of co‐stimulatory receptor agonists holds theoretical appeal. However, clinical development of co‐stimulatory agonists, either as standalone agents or in BsAb formats, for GC is still in its early stages.

#### 3.10.5 Hyperbaric Oxygen Therapy in Combination With PD‐1/PD‐L1 Inhibitors

Hypoxia is a common feature of solid tumors, primarily arising from abnormal blood vessel formation, which leads to inadequate oxygen supply, coupled with increased oxygen demand due to rapid tumor growth [158]. The hypoxic microenvironment triggers the expression of hypoxia‐inducible factor‐1 alpha (HIF‐1α), which activates a range of signaling pathways that promote the expression of collagen genes and connective tissue growth factors. This results in increased collagen synthesis and the formation of a dense extracellular matrix (ECM), which can hinder the effective penetration of macromolecular drugs like anti‐PD‐1/PD‐L1 monoclonal antibodies, limiting their ability to infiltrate tumor tissues. Additionally, hypoxia suppresses the cGAS‐STING pathway, leading to downregulation of type I IFN and NF‐κB, which further dampens the antitumor immune response [159]. Hypoxia also exerts multifaceted immunosuppressive effects: it decreases the expression of MHC molecules on cancer cells, thereby impairing antigen presentation; it upregulates the expression of immune checkpoint molecules such as CTLA‐4 and PD‐1 on relevant cells [160], and it promotes the accumulation or expansion of immunosuppressive cells within the TME [161].

The findings from Liu et al.'s study further support the potential application of hyperbaric oxygen therapy (HBOT) in cancer treatment. Their research demonstrated that HBOT, in vitro, degrades the ECM and remodels the TME, shifting it from an immunosuppressive to an immunostimulatory state. This transformation significantly enhanced the antitumor effects of PD‐1/PD‐L1 inhibitors in stroma‐rich solid tumors. To further explore this potential, our team is conducting a phase Ib/II clinical trial (NCT06742411) evaluating the combination of HBOT with PD‐1 inhibitors and chemotherapy as a first‐line treatment for advanced GC/GEJC.

### 3.11 Targeting Aberrantly Activated Oncogenic Signaling Pathways

**FIGURE 3:**
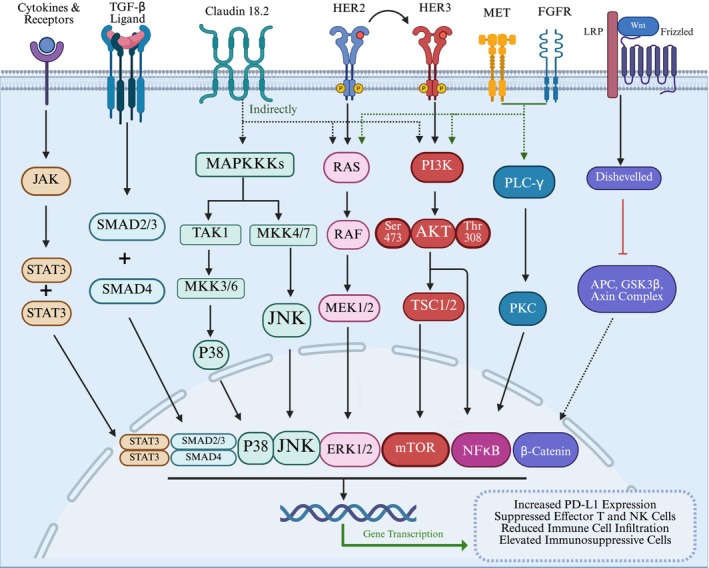
Aberrantly activated oncogenic signaling pathways in GC. This figure illustrates the aberrant activation of key signaling pathways in GC that drive tumor progression and immune evasion. The figure highlights the following pathways: HER2/ERBB2, PI3K/AKT/mTOR, RAS/RAF/MEK/ERK, Wnt/β‐catenin, NF‐κB, JAK/STAT3, and TGF‐β/SMAD. These pathways promote immune escape and tumor growth through various mechanisms, including upregulation of PD‐L1 expression, suppression of effector T cells and NK cells, reduced immune cell infiltration, and increased immunosuppressive cell populations. MET, FGFR, and Claudin 18.2 may indirectly influence these pathways and further contribute to the immunosuppressive TME. These alterations collectively reshape the TME, impairing the efficacy of ICIs and contributing to immune resistance. Created in https://BioRender.com. AKT, Protein Kinase B; ERBB2, Erythroblastic Oncogene B2; ERK, Extracellular Signal‐Regulated Kinase; FGFR, Fibroblast Growth Factor Receptor; HER2, Human Epidermal Growth Factor Receptor 2; JAK/STAT3, Janus Kinase/Signal Transducer and Activator of Transcription 3; MEK, Mitogen‐Activated Protein Kinase Kinase; MET, Mesenchymal‐Epithelial Transition Factor; mTOR, Mammalian Target of Rapamycin; NF‐κB, Nuclear Factor Kappa‐Light‐Chain‐Enhancer of Activated B Cells; PI3K, Phosphoinositide 3‐Kinase; RAF, Rapidly Accelerated Fibrosarcoma; RAS, Rat Sarcoma Virus; TGF‐β, Transforming Growth Factor‐Beta; Wnt/β‐Catenin, Wingless‐Related Integration Site/Beta‐Catenin.

The aberrant activation of multiple oncogenic signaling pathways is a hallmark of cancer, collectively driving malignant progression and actively suppressing antitumor immune responses. These dysregulated pathways create a TME. Key signaling networks implicated include IFNγ/JAK/STAT, WNT/β‐catenin, PI3K‐AKT–mTOR/PTEN, RAS, HER2/ERBB2, NF‐κB, Notch, and TGF‐β [162, 163, 164, 165, 166, 167, 168, 169]. The aberrantly activated signaling pathways in cancer are shown in Figure 3. These pathways facilitate immune evasion through diverse mechanisms, such as upregulating PD‐L1 expression, inhibiting the functions of effector T cells and NK cells, reducing beneficial immune cell infiltration, and enhancing the presence or function of immunosuppressive cells [170, 171, 172]. The aberrant activation of these pathways not only promotes tumor cell proliferation and survival but also modulates the expression of immune‐related genes and alters the composition of the TME. This often leads to a “cold” or non‐inflamed TME, contributing to the reduced efficacy of ICIs and fostering both primary and acquired resistance [166, 173, 174]. Therefore, targeting these aberrantly activated signaling pathways to mitigate their immunosuppressive effects represents a theoretically promising strategy.

### 3.12 AI‐Driven Multi‐Omics for Personalized Immunosensitization in GC

The integration of artificial intelligence (AI) and multi‐omics technologies has emerged as a transformative approach to personalized immunosensitization strategies in GC. The inherent heterogeneity and complexity of GC present significant challenges to traditional treatment paradigms, necessitating the development of more precise, individualized approaches. The rapid advancement of machine learning (ML) algorithms provides a powerful solution, enabling the comprehensive integration and analysis of vast multi‐omics datasets, including genomics, transcriptomics, proteomics, and metabolomics. By deciphering these complex data, researchers can identify novel predictive biomarkers and patient‐specific vulnerabilities, thereby elucidating the intricate interactions within the TME that are critical for enhancing the efficacy of ICIs [175, 176].

For instance, ML models are being effectively employed to construct sophisticated prognostic signatures in GC by integrating multi‐omics data; these signatures, often centered on immune cell infiltration patterns and immune‐related biomarkers, not only predict patient outcomes with high accuracy but can also indicate chemotherapy sensitivity, thereby offering crucial guidance for tailored therapeutic approaches [177]. Furthermore, ML algorithms that integrate radiomics and genomic data can predict spatial heterogeneity and the immune infiltration status within tumors, providing critical insights for patient stratification [178]. To this end, ML models have been developed that translate gene expression signatures into risk scores, which serve as both prognostic indicators and predictors of immunotherapy benefit [179]. Looking forward, continuous advancements in AI and multi‐omics integration will likely enable real‐time, adaptive immunosensitization strategies, further enhancing clinical outcomes for GC patients. Collaborative efforts integrating clinical trials with AI platforms will be pivotal for validating these predictive models and translating them into routine clinical practice.

## 4 Discussion

The advent of PD‐1/PD‐L1 inhibitors has transformed the therapeutic landscape for GC, introducing a novel avenue for harnessing the immune system against tumor cells. However, both primary and acquired resistance remain significant hurdles, underscoring the necessity for combination strategies that enhance the antitumor immune response. Among these, the integration of immunotherapy with chemotherapy has gained particular prominence, becoming the standard of care for HER2‐negative advanced GC. Although numerous studies suggest that chemotherapy augments immune checkpoint blockade, important questions persist regarding the optimal choice of chemotherapy agents, dosing schedules, and treatment duration, all of which demand further clinical investigation. ADCs have also demonstrated potential as immunosensitizers through mechanisms similar to chemotherapy but with the added advantage of a bystander effect, which confines cytotoxicity primarily to the TME, thus potentially reducing collateral damage to immune effector cells.

Beyond cytotoxic regimens, targeting angiogenesis has emerged as another promising strategy. In particular, VEGF/VEGFR blockade can normalize the abnormal vasculature associated with advanced GC, thereby improving immune cell infiltration and reducing immunosuppressive influences within the TME. Early studies combining anti‐VEGF/VEGFR therapies with PD‐1/PD‐L1 inhibitors have shown synergistic effects, improving response rates and survival outcomes. However, the heterogeneity of patients and tumors requires refined biomarker‐driven patient selection and more nuanced dosing strategies. Identifying the subsets of patients who derive the greatest benefit from such combinations remains a critical objective.

Radiotherapy offers yet another avenue for immunosensitization. Beyond direct tumoricidal effects, radiotherapy can remodel the TME by inducing ICD, enhancing antigen presentation, and modulating immune cell populations, with different radiation dosing and fractionation schedules potentially eliciting distinct immunomodulatory outcomes. Although this approach, including innovative concepts like intratumoral dose heterogeneity, has shown promise in other solid tumors, its extensive clinical validation and optimization in GC remain an ongoing research focus.

Additionally, agents targeting distinct molecular and epigenetic pathways—such as DKK1, PARP, LSD1, and HDAC inhibitors—have shown early signals of efficacy in preclinical and early‐phase clinical studies. Although these novel combinations currently lack robust clinical data, their potential to reconfigure the TME and reverse immune evasion justifies further exploration. In this context, AI and ML are increasingly being leveraged to decode complex multi‐omics data, offering valuable insights for biomarker discovery and patient stratification that can further enhance the precision of immunosensitization strategies in GC. Furthermore, emerging evidence suggests that host‐intrinsic factors, such as circadian rhythms influencing treatment timing (chronotherapy) and the patient's psychological state, may also significantly impact ICI efficacy, opening new avenues for personalized optimization that are currently underexplored in GC.

Combination therapies raise concerns about increased immune‐related toxicities, underscoring the importance of balancing efficacy with safety. Moreover, the absence of reliable predictive biomarkers for patient selection continues to hamper clinical decision‐making. Addressing these issues requires large‐scale, randomized controlled trials with well‐defined endpoints. It also necessitates deeper mechanistic studies into the immunobiology of GC and the molecular pathways underlying resistance. Through multidisciplinary collaboration—encompassing oncology, immunology, bioinformatics, and related fields—future research can refine our understanding of the TME, define reliable biomarkers, and implement adaptive trial designs that speed the translation of promising strategies into clinical practice.

In conclusion, while PD‐1/PD‐L1 inhibitors have reshaped the therapeutic landscape for advanced GC, their full potential hinges on the development of rational, combination‐based immunosensitization strategies. As we deepen our mechanistic understanding, refine biomarker‐driven patient selection, integrate emerging technologies such as AI, and carefully balance efficacy with safety, we move closer to a new era of precision immunotherapy—one capable of delivering improved and more durable responses for patients with GC.

## Author Contributions

**Wenke Li:** writing – original draft, writing – review and editing, visualization, investigation, methodology, data curation, project administration. **Menghui Xu:** writing – original draft, writing – review and editing, visualization, investigation, methodology, data curation, project administration. **Mo Cheng:** visualization, investigation. **Jing Wei:** investigation, visualization. **Lin Zhu:** conceptualization, writing – review and editing, supervision. **Yu Deng:** visualization, investigation, software. **Fukun Guo:** investigation, writing – review and editing, software. **Feng Bi:** writing – review and editing, supervision. **Ming Liu:** writing – review and editing, visualization, investigation, software, formal analysis, funding acquisition, project administration.

## Ethics Statement

The authors have nothing to report.

## Consent

The authors have nothing to report.

## Conflicts of Interest

The authors declare no conflicts of interest.

## Data Availability

The authors have nothing to report.
